# Recurrent Peritoneal Inclusion Cyst With Persistent Pelvic Pain Despite Radiologic Improvement: A Management Challenge

**DOI:** 10.7759/cureus.111867

**Published:** 2026-07-01

**Authors:** Kiran Javaid, Riana Tarabocchia, Maria Balabanian, Omar Bellorin-Marin, Rory Ulloque, Rahul Mittal

**Affiliations:** 1 Molecular Biology, Rowan-Virtua School of Osteopathic Medicine, Stratford, USA; 2 Internal Medicine, Holy Name Medical Center, Teaneck, USA; 3 Family Medicine, Holy Name Medical Center, Teaneck, USA; 4 General Surgery, Holy Name Medical Center, Teaneck, USA; 5 Health Informatics, Rutgers University, New Jersey, USA

**Keywords:** abdominal surgery complications, image-guided drainage, pelvic cystic lesion, peritoneal inclusion cyst, recurrent cyst

## Abstract

Peritoneal inclusion cysts (PICs) are benign cystic lesions that most commonly occur in women with a history of prior abdominal surgery, endometriosis, or pelvic inflammatory disease. They arise from the accumulation of peritoneal fluid within adhesions and are associated with high recurrence rates despite available treatment options. We present the case of a 43-year-old woman with a history of prior abdominal surgery who developed recurrent PICs associated with persistent pelvic pain. Imaging demonstrated a large multiloculated pelvic cystic lesion, and pathologic examination confirmed a benign peritoneal inclusion cyst. The patient underwent surgical excision followed by image-guided drainage and alcohol sclerotherapy for recurrent disease. Although follow-up imaging demonstrated interval reduction in cyst size without concerning radiologic features, she continued to experience significant pelvic pain requiring ongoing management. This case highlights the challenges associated with recurrent PICs and demonstrates that radiographic improvement may not necessarily correlate with symptom resolution. Clinicians should consider both imaging findings and patient-reported symptoms when evaluating treatment response and managing recurrent disease.

## Introduction

Peritoneal inclusion cysts (PICs) are benign, multiloculated fluid collections that develop when peritoneal fluid becomes trapped within intra-abdominal or pelvic adhesions. They are uncommon mesothelial-lined cystic lesions that occur predominantly in women of reproductive age and are associated with prior abdominal or pelvic surgery, endometriosis, pelvic inflammatory disease, and other inflammatory conditions that impair normal peritoneal fluid resorption [1-4].

Despite their generally benign behavior, PICs can cause substantial morbidity due to recurrent symptoms and diagnostic uncertainty. Patients frequently present with pelvic pain, abdominal fullness, or symptoms related to mass effect and may mimic ovarian neoplasms and other cystic pelvic lesions on imaging [1,2]. Diagnosis is often established through a combination of clinical history, imaging findings, and, when necessary, histopathologic evaluation [1,2].

Management remains challenging because no standardized treatment guidelines exist. Available treatment options include observation, hormonal suppression, image-guided drainage with or without sclerotherapy, and surgical excision. Although these interventions may alleviate symptoms, local recurrence remains a recognized clinical challenge, and long-term management is often guided by symptom burden and clinical course rather than a standardized treatment algorithm [2-5].

Importantly, the relationship between radiologic findings and symptom severity remains poorly understood. While larger lesions are often presumed to cause greater symptom burden, clinical presentation is highly variable, and symptom severity may not correlate with cyst size or interval radiographic changes [2,5].

We present a case of recurrent PICs in a patient with a history of prior abdominal surgery who experienced persistent and debilitating pelvic pain despite multimodal treatment and an interval reduction in cyst size on imaging. This case highlights the challenges of evaluating treatment response when radiologic improvement does not parallel clinical outcomes and underscores the importance of integrating patient-reported symptoms with imaging findings when managing recurrent PICs.

## Case presentation

A 43-year-old woman with a past medical history of asthma, anemia, arthritis, chronic back pain, gastroesophageal reflux disease, depression, anxiety, uterine fibroids status post hysterectomy (2023), right lower extremity deep vein thrombosis with pulmonary embolism, and migraine headaches presented with severe pelvic pain requiring opioid analgesia for approximately three months. Her surgical history was notable for prior appendectomy, back surgery, shoulder surgery, and diagnostic laparoscopy. She denied tobacco, alcohol, or illicit drug use. Family history was significant for diabetes mellitus, hypertension, cholangiocarcinoma, and breast cancer in her mother. The patient's clinical course is summarized chronologically in Table 1.

**Table 1:** Chronological timeline of the patient's clinical course

Time	Clinical event	Intervention/outcome
Initial presentation	Three-month history of severe pelvic pain	CT demonstrated bilateral adnexal cystic lesions
Initial surgery	Laparoscopic right ovarian cystectomy and left oophorectomy	Peritoneal inclusion cyst identified, evacuated, and confirmed on histopathology
1 month postoperatively	Recurrent suprapubic pain	Recurrent right-sided pelvic cyst; image-guided drainage and alcohol sclerotherapy
2 months postoperatively	Right lower quadrant pain with nausea and vomiting	Aspiration and repeat alcohol sclerotherapy
3 months postoperatively	Persistent pelvic pain	Interval reduction in cyst size on CT despite persistent symptoms; multidisciplinary pain management initiated
January 2026	Follow-up	Persistent 4.7 × 4.0 cm right-sided pelvic cyst; ongoing multidisciplinary pain management

The patient subsequently underwent laparoscopic right ovarian cystectomy and left oophorectomy for persistent pelvic pain. Intraoperatively, extensive adhesions involving the bowel, anterior abdominal wall, pelvic sidewalls, and pelvic structures were identified. A cystic lesion adherent to the peritoneum near the sacrum along the pelvic brim was identified. General surgery was consulted intraoperatively, and fluid was obtained for cytologic evaluation followed by evacuation of the cyst. Histopathologic examination demonstrated a benign peritoneal/mesothelial inclusion cyst. Cytologic evaluation of the cyst fluid was negative for malignant cells. Immunohistochemical staining showed WT-1 and calretinin positivity in mesothelial cells, supporting the diagnosis of a benign mesothelial inclusion cyst. Representative computed tomography (CT) imaging is shown in Figure 1.

**Figure 1:**
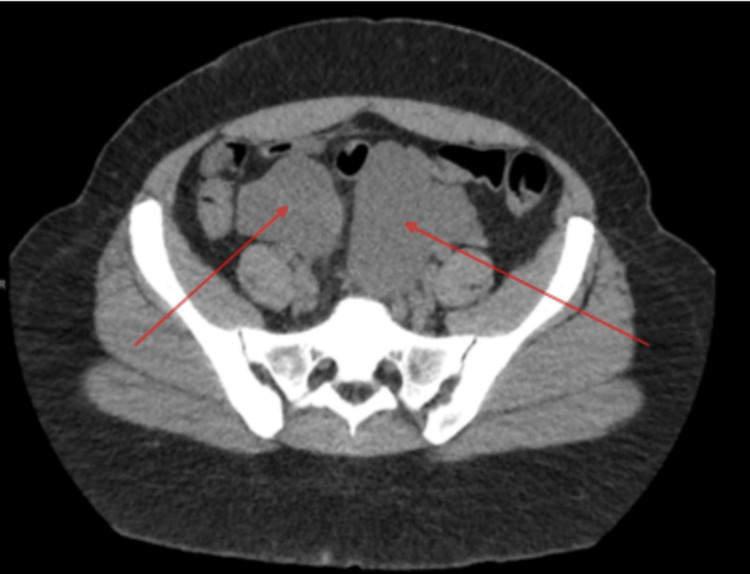
Axial computed tomography image of the pelvis demonstrating bilateral adnexal cystic lesions (red arrows). The left-sided lesion measured 10.0 × 6.5 cm, while the right-sided lesion measured 5.9 × 4.9 cm at presentation

Approximately one month postoperatively, the patient re-presented with worsening suprapubic pain. Imaging demonstrated recurrence of a right-sided cystic pelvic lesion with interval enlargement, measuring 5.8 × 5.6 × 8.4 cm (Figure 2). She subsequently underwent image-guided drainage with alcohol sclerotherapy, resulting in complete evacuation, and was discharged on acetaminophen and tramadol for pain control.

**Figure 2:**
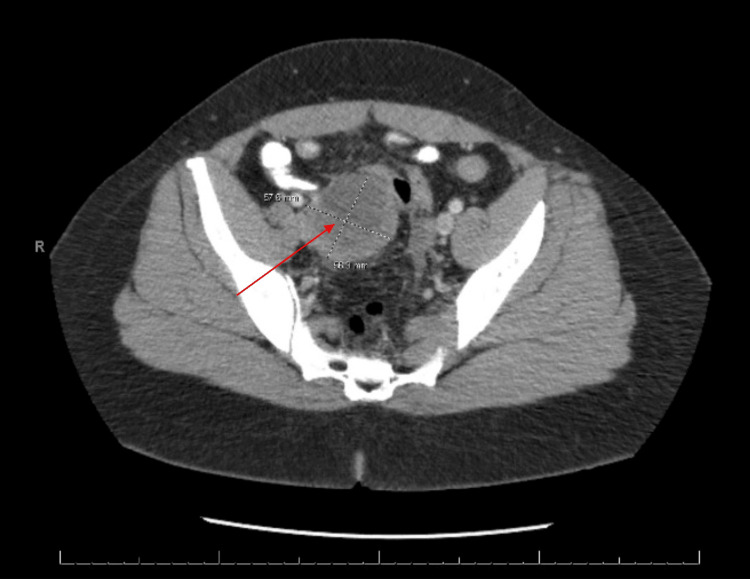
Axial CT image demonstrating recurrence with interval enlargement of a right-sided cystic pelvic lesion measuring 5.8 × 5.6 × 8.4 cm (red arrow)

One month later, she again presented with progressive right lower quadrant pain radiating to the back, associated with nausea and vomiting. Although recurrence of the peritoneal inclusion cyst was initially suspected, imaging findings were also consistent with a hemorrhagic ovarian cyst. She underwent aspiration and alcohol sclerotherapy of this lesion.

Despite these interventions, the patient re-presented one month later with persistent abdominal pain. CT of the abdomen and pelvis demonstrated pelvic cysts without evidence of infection or interval enlargement, with a slight decrease in size compared with prior imaging. Despite interval reduction in cyst size on imaging, the patient continued to experience significant pelvic pain. Given ongoing symptoms despite stable imaging findings, general surgery recommended interventional radiology-guided drainage with temporary catheter placement rather than repeat sclerotherapy to assess symptom improvement. Pain management and gynecology consultations were obtained, and the patient was transitioned to chronic pain management.

On most recent follow-up (January 2026), CT demonstrated a persistent right-sided cystic pelvic lesion measuring approximately 4.7 × 4.0 cm, which was not amenable to further image-guided drainage. Despite the relatively small size of the lesion and absence of concerning radiologic features, the patient continued to experience significant pelvic pain requiring ongoing multidisciplinary management. Representative follow-up imaging is shown in Figure 3.

**Figure 3:**
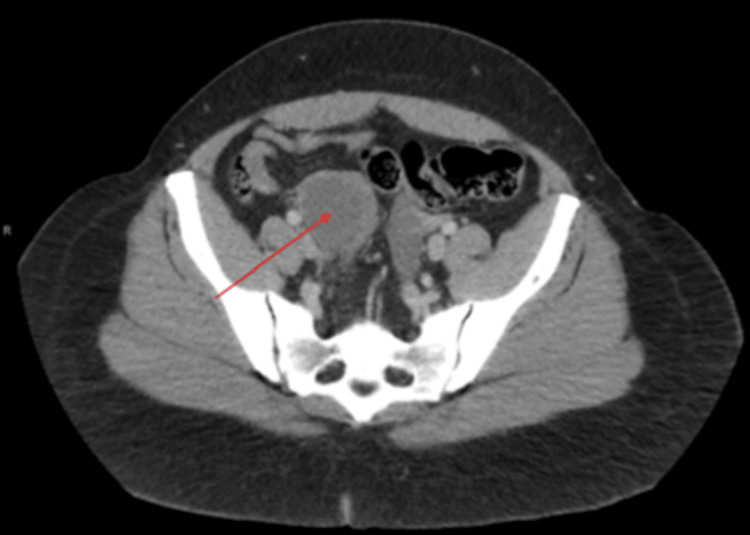
Follow-up axial CT image demonstrating a persistent right-sided cystic pelvic lesion measuring 4.7 × 4.0 cm in the right adnexa (red arrow)

## Discussion

PICs are thought to arise from impaired resorption of physiologic peritoneal fluid within postoperative or inflammatory adhesions, explaining their strong association with previous abdominal and pelvic surgery, endometriosis, and other inflammatory conditions [1,2,4].

Management of PICs remains challenging because no standardized treatment guidelines exist. Available treatment options include observation, hormonal therapy, image-guided aspiration or sclerotherapy, and surgical excision, with treatment selection largely guided by symptom burden, lesion characteristics, and patient-specific factors [2,3,5]. Surgical management may be performed through either laparoscopy or laparotomy. Lee et al. demonstrated that laparoscopic surgery was associated with reduced blood loss, shorter hospital stays, and fewer postoperative complications compared with laparotomy, although recurrence rates were similar between approaches [6].

Despite the availability of multiple treatment modalities, recurrence remains a defining feature of PICs. Reported recurrence rates vary considerably depending on lesion extent, treatment modality, and study population [2,3,7]. More recent clinicopathologic studies suggest that disease progression and disease-related mortality remain exceedingly rare [7]. Vallerie et al. emphasized that PICs are characterized by low mortality but substantial morbidity and suggested that management should focus on symptom relief rather than cure because of the chronic and recurrent nature of the disease [2]. In the present case, recurrence occurred despite surgical excision, image-guided drainage, and sclerotherapy, highlighting the limitations of currently available treatment strategies. Similar challenges in the management of recurrent cystic lesions treated with image-guided sclerotherapy have been described in the interventional radiology literature [8]. The patient's history of multiple prior abdominal and pelvic procedures likely contributed to the development and recurrence of PICs, a relationship that has been described in the literature [2-5].

This case demonstrates that radiographic improvement may not necessarily translate into symptomatic improvement. Despite interval reduction in cyst size and the absence of concerning imaging features, the patient continued to experience significant pelvic pain requiring ongoing management. While treatment response is frequently assessed using imaging findings, this case highlights the importance of incorporating patient-reported symptoms into clinical decision-making and supports a symptom-directed approach to management when symptoms persist despite stable or improving radiologic findings [2,5,8].

## Conclusions

In this patient with recurrent PICs, persistent pelvic pain continued despite surgical excision, image-guided drainage, sclerotherapy, and interval reduction in cyst size on follow-up imaging. This case highlights that imaging findings alone may not adequately reflect treatment response and supports an individualized, symptom-guided approach to the management of recurrent PICs. Further studies are needed to better define the relationship between imaging findings and symptom burden and to optimize management strategies for recurrent disease.
